# The efficacy of sequentially comprehensive treatment based on surgery in the treatment of keloids: a retrospective study

**DOI:** 10.3389/fmed.2024.1492407

**Published:** 2025-01-10

**Authors:** Kun Yang, Mengdong Shi, Shubo Li, Jianning Sun, Ran Huo, Cong Fu

**Affiliations:** ^1^Department of Burn and Plastic Surgery, Shandong Provincial Hospital Affiliated to Shandong First Medical University, Jinan, China; ^2^Department of Burn and Plastic Surgery, Shandong Provincial Hospital, Cheeloo College of Medicine, Shandong University, Jinan, China; ^3^Department of Burn and Plastic Surgery, The People’s Hospital of Huaiyin, Jinan, China

**Keywords:** keloid, sequentially comprehensive treatment based on surgery, clinical effectiveness, recurrence rate, complication

## Abstract

**Purpose:**

The objective of this study is to investigate the clinical efficacy of sequentially comprehensive treatment based on surgery and to furnish clinical evidence for the management of keloids.

**Patients and methods:**

The patients with keloids were retrospectively analyzed who underwent surgery-based sequentially comprehensive treatment at the Plastic Surgery Department of Shandong Provincial Hospital from January 2018 to August 2021. The recurrence rate and incidence of adverse reactions were explored for all the included patients. For patients who were followed up for more than 1 year, the clinical response rate was calculated, and the chi-square test was used to analyze which factors could influence clinical effectiveness. Binary logistic analysis was performed on the factors with statistical differences. For patients with a follow-up time of less than 1-year, paired *t*-test was used to evaluate their Vancouver Scar Scale (VSS) before and after treatment.

**Results:**

A total of 67 patients with 80 keloids were included. The clinical response rate was 81.5% (44/54), the recurrence rate was 15.0% (12/80) and the adverse reaction rate was 4.5% (3/67). The clinical response rate of tumor-type keloids (95.8%) was higher than that of inflammatory-type (70.0%) with a significant difference (*P* = 0.040). After treatment, the color, blood vessel distribution, softness, thickness, and VSS score were all decreased, and the difference was statistically significant (*P* < 0.001).

**Conclusion:**

The sequentially comprehensive treatment based on surgery has a significant curative effect, as well as a low recurrence rate and a low adverse effect rate. The type of keloid has a statistically significant effect on clinical efficacy, and tumor-type keloids are more suitable for sequentially comprehensive treatment based on surgery.

## Introduction

Keloid, also known as “crab claw,” is an overgrown pathological scar tissue that spontaneously forms or is secondary to skin trauma. It usually shows that the lesion exceeds the scope of the original skin lesion, higher than the surface of the surrounding normal skin, and presents a continuous growth of nodular, cord-like, or sheet-like swelling tissue with tough and congestive texture, which is prone to occur in the anterior sternum, upper back, and upper arm triangle (1). Keloids are characterized by several distinct pathological features, including: (1) enhanced fibroblast proliferation, marked by an active mitotic phase; (2) a noticeably thickened and flattened epidermal layer; (3) a distinctive advancing margin within the dermis; (4) disorganized and thick hyalinized collagen bundles, which are a prominent feature of the dermis, resulting in the obliteration of the papillary-reticular junction; (5) elevated cellular density within the dermal compartment; (6) signs indicative of inflammation; (7) fluctuating expression levels of α-smooth muscle actin (α-SMA) (2). These characteristics together define the unique morphological and pathological landscape of keloids. The incidence of keloids is affected by racial ethnicity, higher prevalence of keloids in Asian populations compared to Caucasians (3, 4).

Keloid, a prevalent condition in the field of plastic surgery, exhibits notable resistance to treatment and high rates of recurrence (5). Surgical and non-surgical interventions are the primary therapeutic options for scar treatment. Among the non-surgical treatment methods include the application of silicone, local pressure, local injection of drugs, cryotherapy, laser, etc (6). Previous research has indicated that the recurrence rate of surgical excision alone can range from 45 to 100%, with recurrent scars being significantly larger in volume compared to the initial scar tissue (6). Currently, with the complex formation mechanism of keloids and the diverse treatment means, there is still a lack of uniform standard comprehensive treatment measures for keloids (7). This study aims to provide more clinical evidence for management of keloids by retrospective analysis of the clinical effects of patients with keloids who have received sequentially comprehensive treatment based on surgery.

## Materials and methods

### Patient collection

Patients with keloids who attended the Department of Plastic Surgery of Shandong Provincial Hospital for sequentially comprehensive treatment from January 2018 to August 2022 were collected for this study. Patients who met the following criteria were excluded:(1) patients with postoperative pathological findings showing non-keloid lesions (2) patients who did not undergo surgical excisional treatment (3) women during pregnancy or lactation.

All patients received sequentially comprehensive treatment based on surgery. Prior to treatment initiation, patients underwent preoperative assessments including blood routine examination, coagulation function evaluation, and electrocardiography, among others, to rule out any surgical contraindications. Additionally, patients provided informed consent by signing a consent form for both medical intervention procedure and inclusion in publication.

### Treatment methods sequentially comprehensive treatment

(1) Surgical resection (workflow diagram shown in Supplementary Figure 1): Different surgical techniques were selected based on the location, size, and distribution of the keloids. In cases where the keloid was located in the ear, the core excision method was performed on the lesion, while the remaining keloids were completely excised. Following excision, the surgical area was rinsed sequentially with glucocorticoids, 5-fluorouracil, and saline. For keloids with lengths ranging from 2.0 to 10.0 cm and widths of ≤5.0 cm, the tension-reducing suture was directly applied after excision. In instances where the keloids exceeded 10.0 cm in length and 5.0 cm in width or where the local tension was high, flap transfer or skin graft was chosen to cover the resulting trauma after complete excision of the keloids in a single procedure.

(2) Intraoperative and post-operative administration of hormonal and antitumor chemotherapeutic agents: In the case of partially resected keloids or patients ineligible for postoperative radiation therapy, immediate injection of triamcinolone and 5-fluorouracil were administered at the incision site following surgery. The injection was prepared by mixing 2 ml of triamcinolone (10 mg/ml) with 0.3 ml of 5- fluorouracil stock solution(25 mg/ml) and 2.0 ml of 2% lidocaine.

(3) Post-operative radiation therapy: Postoperative radiation therapy was administered promptly, within 24 h following the surgical procedure. The treatment involved the application of superficial X-rays, with an irradiation range extending approximately 5 mm outward from the surgical site in all directions. A distance of 15 cm was maintained during irradiation, and lead plates were employed to safeguard the surrounding skin. The prescribed regimen consisted of 5 daily treatments, each delivering a single irradiation dose of 4 Gy. The cumulative irradiation dose did not exceed 20 Gy.

(4) Wound healing stage: The recommended course of action includes the application of topical silicone medication for a minimum of 6 months following wound healing. Additionally, tension-reducing treatment should be implemented involving the gentle pulling of normal skin tissues on both sides of the vertical incision line to alleviate tension in the surrounding skin. Regular monitoring is essential, and if any signs of recurrence such as incision bulging, itching, or pain arise, the administration of triamcinolone and 5-fluorouracil via injection into the edges of the incision and abnormal scar tissues may be considered. The injection was prepared by mixing 5.0 ml of triamcinolone (10 mg/ml) with 0.6 ml of 5- fluorouracil stock solution (25 mg/ml) and 2.0 ml of 2% lidocaine. Treatment intervals for injections in recurrent patients were 1 month until the incision returned to flatness and softness, requiring 3–5 treatments. Intervention in the early stages of recurrence was recommended.

### Clinical efficacy evaluation

**FIGURE 1:**
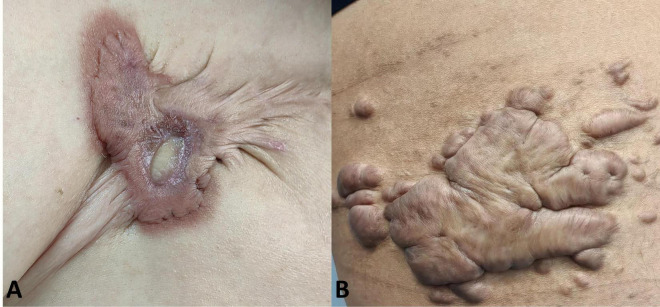
Typical clinical manifestations of two subtypes of keloids. **(A)** Inflammatory-type keloid, distinguished by swift peripheral infiltration and pronounced congestion; **(B)** tumor-type keloid, characterized by prominently bulging and appearing less congested or darker.

The clinical data of patients who met the inclusion criteria were collected, including age, gender, site, size (A small-sized keloid is defined as having a diameter less than 2.0 cm, while a medium-sized keloid is characterized by a length ranging from 2.0 to 10.0 cm and a width less than 5.0 cm, a large-sized keloid is identified by a length exceeding 10.0 cm and a width equal to or greater than 5.0 cm.) and thickness of keloid, family history, etiology, type (inflammatory-type or tumor-type, inflammatory-type keloid is distinguished by swift peripheral infiltration and pronounced congestion, on the other hand, tumor-type keloid is characterized by prominent protrusion from the skin surface without significant congestion, typical clinical manifestations are shown in Figure 1) (8), surgical procedure, whether radiotherapy was given after surgery, adverse reactions during treatment, whether anti-scar treatment was given after surgery, incision scar characteristics (vascularity, thickness, pliability and pigmentation), whether recurrence, time of recurrence and clinical manifestations.

(1) The clinical effective rate was assessed for patients with a follow-up period exceeding 1 year, with treatment outcomes categorized as cured, markedly effective, and ineffective. The evaluation criteria are presented in Table 1. Total clinical effective rate = (number of cured cases + number of markedly effective cases) / total number of cases × 100%.

**TABLE 1:** Evaluation criteria.

Outcome	Clinical manifestations
Cured	Symptoms such as pain and itching disappear, and there are ordinary scars on the surgical site. Follow up for at least 1 year, the skin lesions have softened and there are no signs of recurrence such as induration or thickening
Markedly effective	Occasionally, patients experience symptoms such as pain and itching, alongside the presence of mild scar hyperplasia in the surgical region, most skin lesions have softened without significant thickening, and have not worsened after at least 1 year of follow-up.
Ineffective	Patients experience obvious symptoms such as pain and itching, and keloids reappear at the surgical region.

(2) The effectiveness of the treatment was evaluated using the Vancouver Scar Scale (VSS) for patients with a follow-up period of less than 1 year (9). The VSS assigns a total score of 15, to evaluate the vascularity (0 = normal, 1 = pink, 2 = red, and 3 = purple), pigmentation (0 = normal, 1 = hypopigmentation, 2 = hyperpigmentation), pliability (0 = normal, 1 = supple, 2 = yielding, 3 = firm, 4 = banding, 5 = contracture) and height (0 = flat, 1 = 0–2 mm, 2 = 2–5 mm and 3 > 5 mm), with higher values indicating greater severity of scar proliferation.

### Statistical method

Counting data were expressed as frequency and percentages (%), and grade data were subjected to analysis using the rank-sum test. The statistical methods utilized for analysis and processing encompassed the chi-square test and paired *t*-test, with statistical significance defined as *P* < 0.05. In the aforementioned analysis findings, both the factors exhibiting statistically significant differences and the factors that, although not statistically significant, are deemed clinically relevant to the dependent variable, have been incorporated as independent variables and subjected to multivariate logistic regression analysis.

## Results

### Patient characteristics

A total of 67 cases were included in this study, including 24 male patients and 43 female patients, with a male-to-female ratio of 1:1.79. 80 keloids were found in 67 patients, including 26 (32.50%) in the ear, 15 (18.75%) in the anterior chest, 15 (18.75%) in the back and shoulders, 7 (8.75%) in the abdomen, 7 (8.75%) in the face, 3 (3.75%) in the upper extremities, 3 (3.75%) in the knees, 2 (2.50%) in the perineum, and 2 (2.75%) in feet. The 80 included keloids were classified according to their origins. 38 (47.5%) keloids were the inflammatory-type and 42 (52.5%) keloids were the tumor-type. As shown in Table 2. 67 patients underwent surgical resection of the keloids, 45 patients completed 5 radiation treatments. 20 patients did not undergo radiation therapy, and two patients discontinued after receiving radiation therapy because of discomfort.

**TABLE 2:** Demographic of patients’ keloid characteristics.

Demographic of patients	Number of patients (%)
**Gender**	
Female	24 (36%)
Male	43 (64%)
**Age**	
Adult	56 (83%)
Child	11 (17%)
**Follow-up time**	
≥1 year	47 (70%)
<1 year	20 (30%)
**Location**	
Head and neck	33 (41%)
Trunk	39 (49%)
Limb	8 (10%)
**Type**	
Tumor-type	42 (52%)
Inflammatory -type	38 (48%)
**Etiology**	
Surgery	18 (22%)
Acne	17 (21%)
Trauma	31 (39%)
Spontaneous	14 (18%)
**Family history**	
Yes	4 (5%)
No	76 (95%)
**Rupture or not**	
Yes	11 (14%)
No	69 (86%)

### Clinical effective rate and analysis of influencing factors

#### Clinical effective rate

47 patients were followed up for more than 1 year after surgery, with a total of 54 keloids. Among the total cases observed, 30 (55.6%) were cured, 14 (25.9%) were effective, and 10 (18.5%) were deemed non-responsive. Consequently, the clinical effective rate was determined to be 81.5%.

#### Analysis of influencing factors

(1) The chi-square test, as evidenced in Table 3, revealed that there was no statistically significant difference (*P* > 0.05) among the influencing factors of gender, site, etiology, family history, preoperative rupture, size, and clinical efficiency. However, there were significant differences (*P* = 0.040) in the clinical efficiency between different types of keloids. Specifically, the inflammatory-type of keloid exhibited an efficiency of 70.0%, which was notably lower than the efficiency observed in the tumor-type keloid (95.8%). Logistic regression model was used to exclude confounding factors.

**TABLE 3:** Impact factors of clinical effective rate.

Variable	Effective *N* = 44	Ineffective *N* = 10	Effective rate	*P*-value	*P*-adj
**Sex**					
Male	13	3	81.3%	0.640	
Female	31	7	81.6%		
**Location**					
Ear	23	2	92.0%	0.240	
Head and neck	5	2	71.4%		
Chest and shoulder	10	3	76.9%		
Abdomen	4	2	66.7%		
Limb	2	1	66.7%		
**Type**					
Inflammatory-type	21	9	70.0%	0.040*	0.040
Tumor-type	23	1	95.8%		
**Etiology**					
Trauma	19	3	86.4%	0.744	
Surgery	9	2	81.8%		
Acne	10	4	71.4%		
Spontaneous	6	1	85.7%		
**Family history**					
Yes	2	1	66.7%	0.47	
No	42	9	82.4%		
**Rupture or not**					
Yes	4	2	66.7%	0.31	0.582
No	40	8	83.3%		
**Size**					
Small-sized	18	3	85.7%	0.877	
Medium-sized	22	6	78.6%		
Large-sized	4	1	80.0%		

*P < 0.05.

(2) The statistical analysis conducted in this study revealed that preoperative rupture did not exhibit a significant association with clinical effectiveness. Nevertheless, it was observed that follow-up time and whether to rupture were influential factors impacting clinical effectiveness. Consequently, the logistic regression model incorporated the following independent variables: type, follow-up time, and whether to rupture, as depicted in Table 4. The logistic model obtained in the final analysis demonstrated statistical significance, χ2 = 8.163, *P* = 0.043. Notably, among the three independent variables incorporated in the model, a statistically significant distinction in clinical effectiveness was observed between the various types of keloids. Specifically, the tumor-type keloid exhibited a clinical efficiency that was 9.7 times greater than that of the inflammatory-type keloid.

**TABLE 4:** The results of the multivariate logistic analysis.

	B	SE	Wald	Freedom	Significance	Exp(B)	95% CI for Exp (B)
Followup time	−0.030	0.034	0.776	1	0.378	0.970	0.907–1.038
Type	2.272	1.104	4.236	1	0.040	9.704	1.115–84.483
Rupture	−0.589	1.069	0.303	1	0.582	0.555	0.068–4.514
Constant	1.792	1.058	2.869	1	0.090	5.999	

#### Vancouver scar scale score

A total of 20 patients, each with less than 1 year of postoperative follow-up, were included in the study. Among these patients, a total of 26 keloids were observed. The paired *t*-test was employed to analyze the scores obtained before and after treatment. The statistical analysis revealed a reduction in vascularity, thickness, pliability, pigmentation, and VSS scores following treatment and the difference was statistically significant (*P* < 0.001). These findings are presented in Table 5.

**TABLE 5:** Vancouver Scar Scale scores before and after treatment.

Variable	Pre-treatment	Post-treatment*	*P-*value
Pigmentation	2.4 ± 0.50	1.2 ± 0.37	<0.001
Vascularity	2.4 ± 0.57	1.2 ± 0.51	<0.001
Pliability	3.3 ± 0.63	1.3 ± 0.62	<0.001
Thickness	3.7 ± 0.47	1.5 ± 0.95	<0.001
VSS scores	11.8 ± 1.2	5.3 ± 1.9	<0.001

*The median follow-up time was 9 months, ranging from 6 to 11 months.

#### Recurrence rate

**FIGURE 2:**
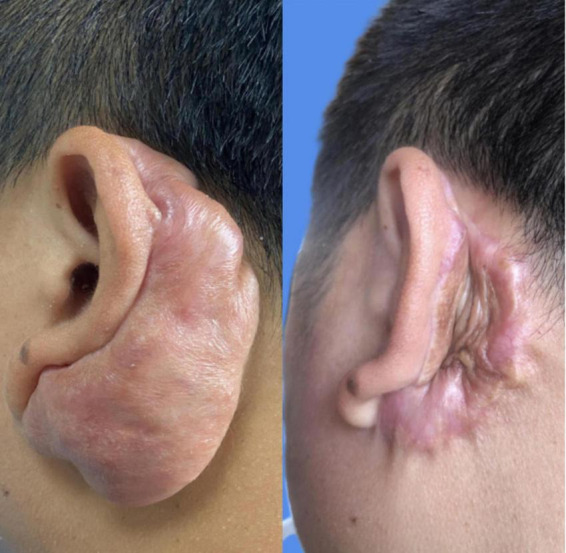
The recurrence of keloid behind the auricle.

A total of 67 patients, with 80 keloids, were enrolled in the study. Out of these, 12 keloids experienced recurrence, resulting in a recurrence rate of 15.0% (12/80). Specifically, the distribution of these recurrences was as follows: 3 in the chest and back, 3 in the ear, 3 in the abdomen, 2 in the face, and 1 in the elbow joint. For instance, as depicted in Figure 2, a patient diagnosed with an auricular keloid underwent surgical excision followed by postoperative radiotherapy, subsequently experiencing recurrence after a duration of 11 months.

#### Adverse reactions

**FIGURE 3:**
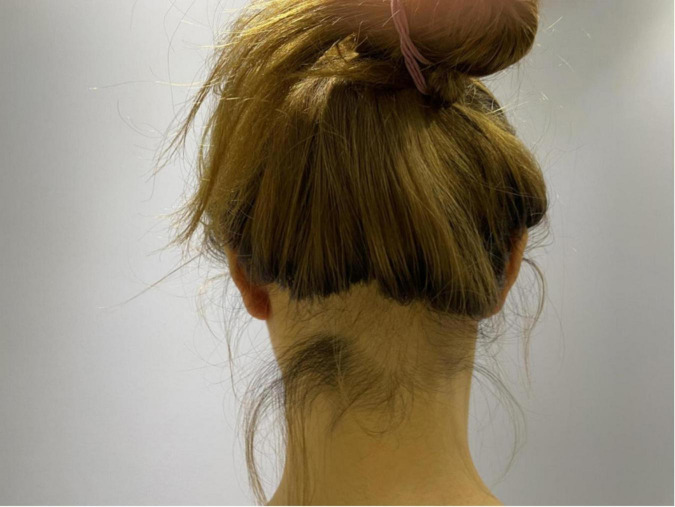
Local alopecia after radiotherapy.

Among the 67 patients observed, there were three instances of adverse reactions, resulting in an adverse reaction rate of 4.5% (3/67). One patient experienced ulceration and pus at the incision site following stitch removal, which was successfully treated through active dressing changes. However, the patient subsequently experienced a recurrence of keloid in the operated area. Another patient reported an allergic reaction characterized by generalized itching and rash after the first radiotherapy session. Following the patient’s refusal of further radiotherapy, the allergy improved without needing specific treatment. A patient with bilateral earlobe keloids experienced the onset of craniocervical alopecia over a month following the completion of five radiotherapy sessions, at the 10-month follow-up, the hair in the alopecia region had regrown without any abnormal hair quality, as depicted in Figure 3.

## Discussion

Keloids can cause localized impairment in both aesthetic appearance and functional abilities. This progression is frequently accompanied by various symptoms including pain, pruritus, and ulceration (1, 10–12). When solely employing the monotherapy for keloid treatment, a frequent occurrence of relapse is observed, necessitating the utilization of multiple treatment approaches in combination. Consequently, the author advocates for a surgery-based sequentially comprehensive treatment. By allowing doctors to make timely treatment decisions based on individual patient conditions, the dynamic treatment strategy facilitates the achievement of the most favorable expected outcomes in clinical practice (13). Sequential therapy aims to optimize the outcome over time by addressing different stages of the treatment process with specific strategies (14).

The sequentially comprehensive treatment can be categorized into three distinct phases: the initial phase involves clarifying the diagnosis and enhancing patient education on the disease to prevent keloid infection and rupture. the subsequent phase primarily focuses on lesion removal and facilitating wound healing; and the final phase pertains to maintenance, primarily aiming to prevent keloid recurrence. During the surgical procedure, it is imperative for the surgeon to focus on the tension-reduction operation (15, 16). The application of glucocorticoids, 5-fluorouracil, and saline in a sequential manner to rinse the surgical site before wound closure has been shown to be an effective strategy for decreasing the occurrence of postoperative recurrence (17). In cases where partial resection of keloid has been performed or when postoperative radiation therapy is not feasible, immediate administration of triamcinolone acetonide injection and 5-fluorouracil injection at the incision site has been implemented (18, 19). Patients in this study underwent radiation therapy within 24 h after their surgery, as previous research has indicated that the most favorable outcomes are obtained when radiation therapy is administered within this timeframe (20–22). The patients included in this study were treated with superficial X-rays. For children who have experienced a recurrence or have rapidly and severely developed keloids, the decision to utilize radiation therapy can be made in consultation with a radiologist (23). Emphasizing postoperative care, such as regular dressing changes, can effectively minimize inflammatory response and subsequently decrease the likelihood of recurrence (24). Additionally, in cases where postoperative skin exhibits signs of recurrence such as erythema, pruritus, and pain, the injection of glucocorticoids into both sides of the incision margin and abnormal scar tissue can be considered.

A total of 67 patients were included in the study, with a total of 80 keloids. Among them, 47 cases were followed up for more than 1 year, with a total of 54 keloids. 30 (55.6%) were cured, 14 (25.9%) were markedly effective, and 10 (18.5%) were deemed ineffective. The clinical effective rate was 81.5%. A statistically significant distinction in the efficacy of various types of keloids (*P* = 0.040) persisted, suggesting that tumor-type keloid exhibits superior effectiveness compared to inflammatory-type keloid (25). The findings of this study demonstrate that tumor-type keloid exhibits superior clinical efficacy for a surgery-based sequentially comprehensive treatment. Consequently, in clinical treatment, surgical resection can be regarded as the preferred treatment modality for tumor-type keloid.

Meanwhile, in this study, the effective rate of keloids in the ear was found to be as high as 92%, which aligns with the findings reported in the literature regarding the combination of keloid core excision of the ear and radiotherapy (21, 26). This suggests that the treatment approach focusing on keloid core excision of the ear in conjunction with radiotherapy not only yields a high therapeutic efficacy but also optimizes the restoration of the natural aesthetic morphology of the patient’s ear.

Three patients reported adverse reactions during the treatment process, one patient had ulceration and pus at the incision after suture removal, although the wound healed well after active treatment, the keloid in the operated area recurred more than 2 months after the operation; Two additional adverse reactions were associated with radiotherapy. One patient experienced an allergic reaction characterized by generalized itchiness and rash following the initial radiotherapy session. This symptom spontaneously resolved after the patient declined further radiotherapy. Another patient, who had bilateral earlobe keloids, developed alopecia in the craniocervical region over a month after completing five radiotherapy treatments. However, at the 10-month follow-up, the hair in the alopecia region had regrown without any abnormal hair quality. This particular complication has not been documented in the pertinent academic literature thus far, and it is anticipated that it will garner clinical attention. Adverse reactions associated with radiotherapy are contingent upon therapeutic variables, including the treatment site, radiation modality, radiation dosage, cumulative dosage, fractionation, and overall treatment time (27).

This study reveals that patients with a prior or familial history of keloid should minimize exposure to avoidable trauma or surgery, while promptly addressing skin issues like acne and festering infection to mitigate the likelihood of keloid occurrence. Nevertheless, in clinical practice, it was found that patients’ compliance with glucocorticoids injection was poor, so few patients after discharge completed regular hormone injection treatment. In the subsequent investigation, it is imperative to enhance patient education, secure improved patient cooperation, and delve deeper into the integrated utilization of surgery, radiotherapy, and hormone injection.

This topic exhibits several limitations. The present study adopts a retrospective analysis approach, which introduces a potential recall bias. Despite adhering to rigorous inclusion and exclusion criteria, ensuring the homogeneity and completeness of the follow-up data remains challenging. Consequently, a multicenter randomized controlled study with a larger sample size is needed to explore the clinical value of dynamic sequential therapy based on surgery.

## Conclusion

This study indicate that this sequentially comprehensive treatment based on surgery exhibits notable clinical effectiveness, and a significant reduction in the VSS score, while also demonstrating a low recurrence rate and occurrence of adverse reactions. Furthermore, it has been found that the type of keloid has a statistically significant effect on clinical effectiveness. It’s worth noting that the comprehensive treatment process should emphasize thorough patient-doctor communication and shared decision-making. Prospective, controlled studies to systematically compare the safety and efficacy of surgery-based versus non-surgical comprehensive treatments are needed in the future.

## Data Availability

The raw data supporting the conclusions of this article will be made available by the authors, without undue reservation.

## Supplementary material

Supplementary Figure 1 Workflow diagram of sequentially comprehensive treatment based on surgery.
