# Supplementary figures and images for: The efficacy of sequentially comprehensive treatment based on surgery in the treatment of keloids: a retrospective study
Source: Front Med (Lausanne). 2025 Jan 10;11:1492407. doi: 10.3389/fmed.2024.1492407 (PMC11757127; doi:10.3389/fmed.2024.1492407)

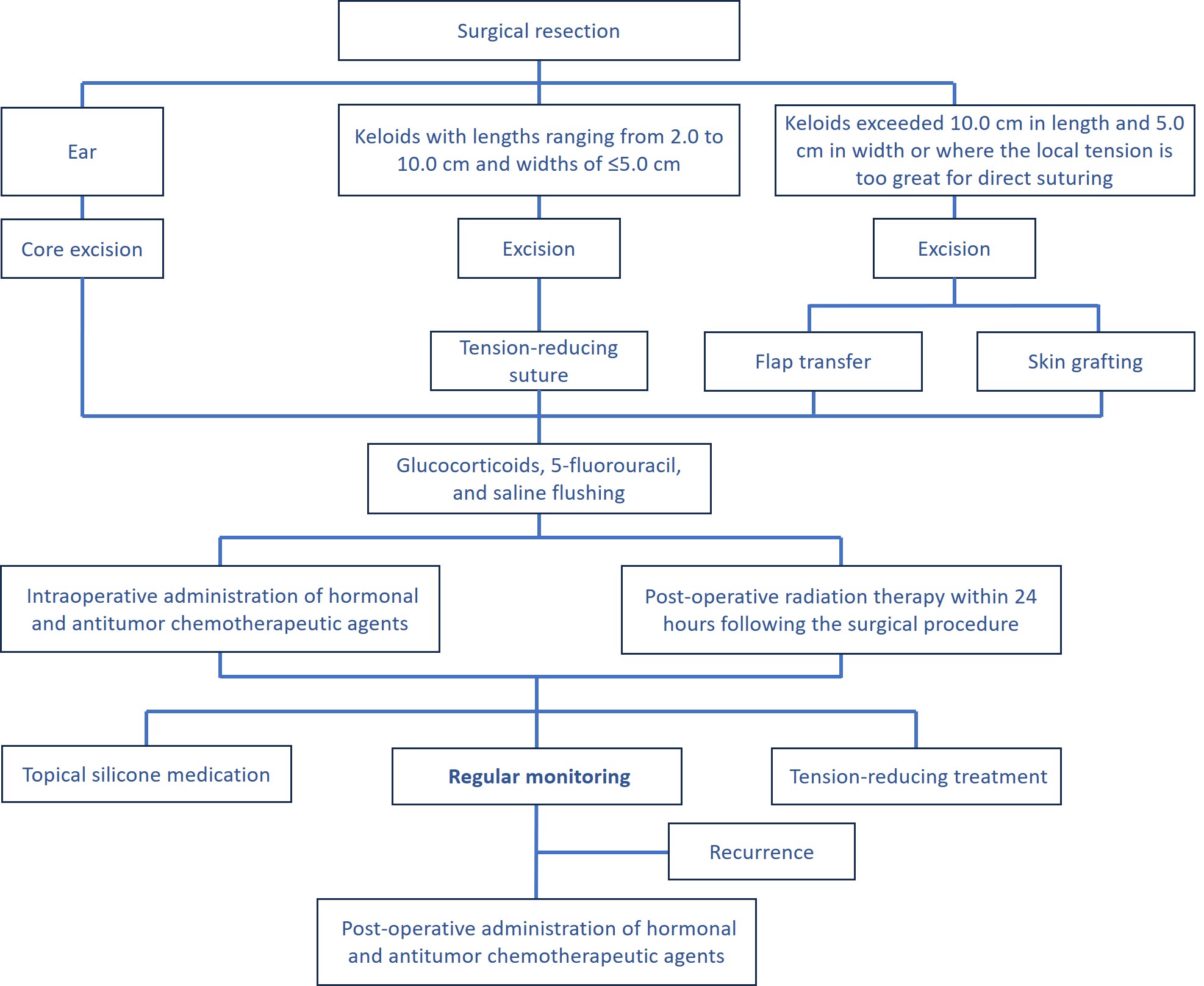

Supplement: Supplementary Figure 1 — Workflow diagram of sequentially comprehensive treatment based on surgery. [file Image_1.jpeg]
